# Climate change adaptation responses among riparian settlements: A case study from Bangladesh

**DOI:** 10.1371/journal.pone.0278605

**Published:** 2022-12-07

**Authors:** Walter Leal Filho, G. M. Monirul Alam, Gustavo J. Nagy, Mohammad Mahbubur Rahman, Sajal Roy, Franziska Wolf, Marina Kovaleva, Mustafa Saroar, Chunlan Li

**Affiliations:** 1 Research and Transfer Centre “Sustainable Development and Climate Change Management”, Hamburg University of Applied Sciences, Hamburg, Germany; 2 Department of Natural Sciences, Manchester Metropolitan University, Manchester, United Kingdom; 3 Faculty of Agricultural Economics and Rural Development, Bangabandhu Sheikh Mujibur Rahman Agricultural University, Gazipur, Bangladesh; 4 School of Commerce, University of Southern Queensland, Toowoomba, Queensland, Australia; 5 Instituto de Ciencias Ambientales y Ecología, Facultad de Ciencias, Universidad de la República, Udelar, Montevideo, Uruguay; 6 Department of Sociology, Lancaster University, Lancaster, United Kingdom; 7 Centre for Social Impact, UNSW Business School, University of New South Wales, Sydney, Australia; 8 School of Social Sciences, UNSW Sydney Campus, Sydney, Australia; 9 Department of Urban and Regional Planning, Khulna University of Engineering & Technology (KUET), Khulna, Bangladesh; 10 Center for Geopolitical and Strategic Studies and Institute for Global Innovation and Development, East China Normal University, Shanghai, China; 11 School of Urban and Regional Sciences, East China Normal University, Shanghai, China; Universitat Autonoma de Barcelona, SPAIN

## Abstract

As transition areas between aquatic ecosystems and the adjacent terrestrial ones, riparian regions are highly exposed to coastal climate hazards. This article describes how climate change and extreme weather impact vulnerable riparian communities and settlements. The analysis is done by reviewing past research and empirical case studies from riparian rural communities of the impact zone of the Sundarbans in Bangladesh, the world’s most extensive mangrove forest. The article discusses the climate-related impacts on households through a Severity Index of Vulnerability and assesses the adaptation responses they may pursue. The principal climate-related vulnerabilities and impacts due to increases in temperature, storm surges, sea flooding, and sea-level rise are seawater intrusion and riverbank erosion. Many households have adopted several autonomous reactive adaptation strategies rather than planned ones, to cope with these impacts. However, government organisations and NGOs provide less than optimal technical and financial support to households for planned and anticipatory adaptive responses. The main barriers to adaptation were the high cost of improved crop varieties, inadequate agricultural extension services, and a lack of knowledge on effective climate adaptation. The restoration of the mangrove ecosystem may increase its resilience and, among other things, make local communities less exposed. The article also presents some adaptation measures proper to reduce the climate-related vulnerability of riparian settlements.

## 1. Introduction: Riparian zones

**Fig 1:**
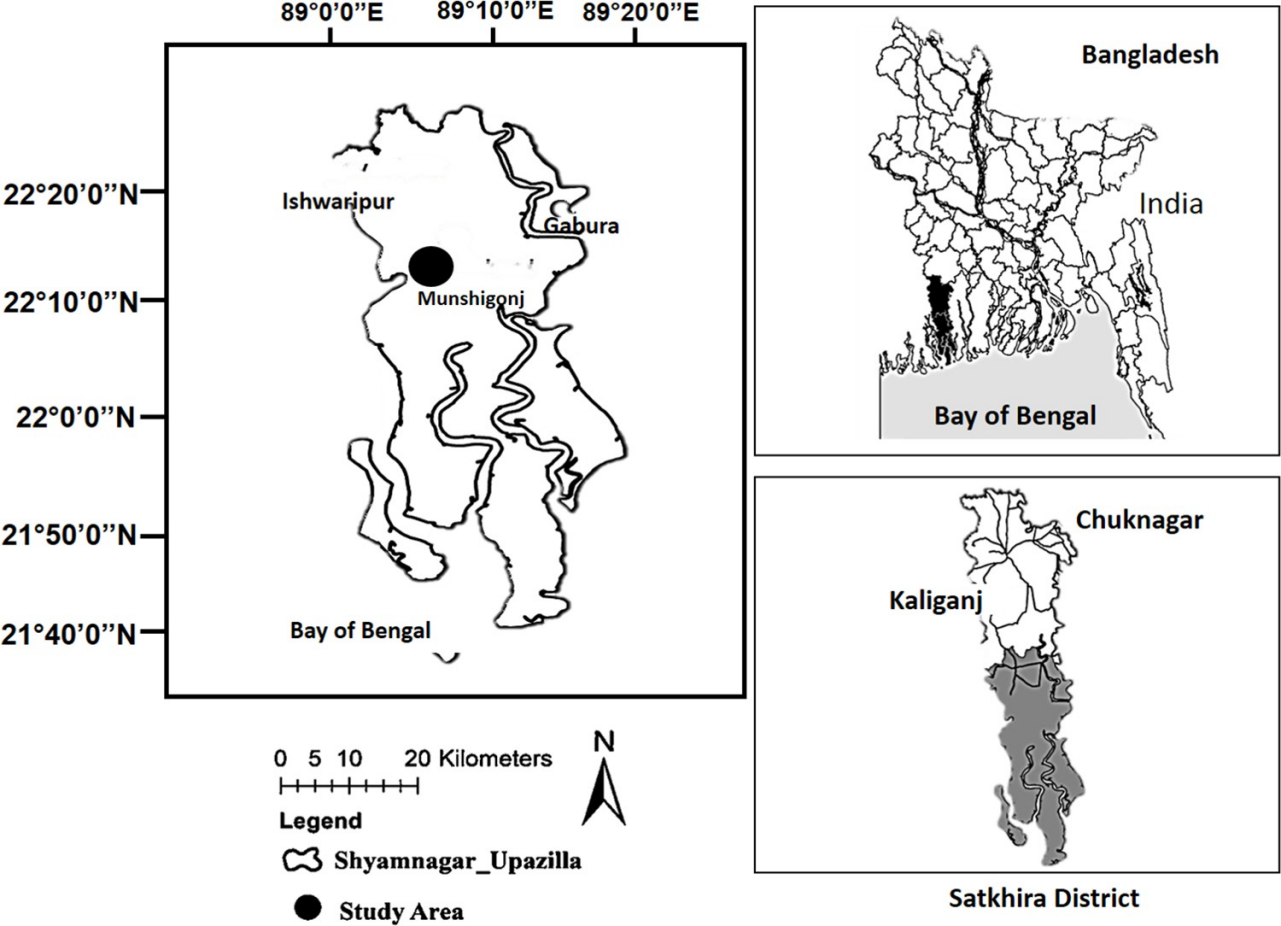
The southwestern coastal region of Bangladesh.

Riparian landscapes are regions along rivers and water flows, including their natural resources and regimes [1]. Riparian areas are transitional zones or ecotones with aquatic and terrestrial highlands ecosystem characteristics (Fig 1) [1]. Despite the provision of eco-services, riparian zones (RZs) remain a frontline for ecosystem studies and those aimed at the conservation, restoration, and management of terrestrial-aquatic landscapes [2, 3]. RZs are landscapes built within flowing waterways at the intersection of land and freshwater ecosystems, contributing substantially to the region’s biodiversity [4]. RZs are also defined as riparian vegetation, corridors, or galley woodlands [3].

RZs are the habitat for many biotic communities found along the shores and banks of rivers and streams, along the boundaries of lakes, reservoirs, springs, bogs, and meadows, and on the border between terrestrial and aquatic habitats [5, 6]. Some of the critical biotic and abiotic characteristics of RZs are:

- highly variable flooding, characterised by a landscape of vegetation and barren river ecosystems functioning as hierarchical habitats [7].
- different biological populations with species benefiting from abundant water and nutritional resources [2, 8].

RZs offer a variety of essential eco-services such as habitat for biodiversity; carbon storage; flood mitigation and bank stability; cooling and growing moisture for microclimate management; and water and nutrient cycling [2, 3, 9, 10].

Many towns in the world are adjacent to rivers [11], which are vital to them. For example, the Ganges River system contributes considerably to supporting local agriculture, livestock farming, fisheries, tourism, river trade, and transport to the livelihood, food, and nutritional well-being of around one-third of the population in India, and two-thirds of the Bangladeshi population [12].

Riparian ecosystem categorisation involves parameters such as climate, humidity, a succession of plants, vegetation, and streams [5]. Riparian areas should not be confused with biomes. Biomes are the most basic forms of ecological species, primarily influenced by their environment or climate [13]. Biomes are exemplified by rainforests, savannas, chaparral, grasslands, and deserts. Some biomes have a riparian zone along with their freshwater bodies’ surface [1].

Likewise, riparian ecosystems’ habitats are unique and distinct, primarily because of these areas’ extra water, soil, and vegetation features. The confluence of these elements in the RZs influences higher moisture content than the terrestrial dyer ecosystem located adjacent (at the upper catchment) to the RZs [14]. Conversely, in comparison to the nearby (or located downstream) aquatic ecosystems that are inundated with water year-round or for extended periods, riparian environments are considerably drier.

RZs have undergone significant human-induced changes since the advent of civilization [2, 15], which have impacted trophic networks at each stage [2, 16]. For example, agricultural activities such as grazing and livestock rearing utilise vast nutrient-rich substrates of floodplains [17, 18]. Several activities such as building dams, water extraction for irrigation, residential and commercial use, and the channeling and destruction of vegetation also affect riparian environments [2, 11]. Likewise, streams have been modified to act as transport, tourism, aquaculture, agrarian, and urban development corridor in riparian zones [2]. Other activities, like the invasion of exotic species and erosion, also affect riparian zones [11].

Riparian areas are therefore affected by a wide range of human activities. As a result, resource managers face the challenge of detecting impacts over a vast region and, if possible, improving both the quality of the watercourse and the associated vegetation [19]. Several scholars and researchers have suggested different approaches to preserving riparian areas; for instance, Hunter et al. [20] and González et al. [2] proposed a conservation plan centred on five critical activities: awareness, inventory, security, environmental management, and restoration.

Bangladesh is a riparian country on the Ganges–Brahmaputra-Meghna (GBM) watershed [21]. More than 230 rivers and tributaries flow through Bangladesh, carrying billion of tons of sediment and releasing enormous amounts of water annually to the Bay of Bengal [22]. Hence, the increased monsoonal flows strongly erode the riverbank in the GBM deltaic region covering southwest riparian mangrove swamp as well in Bangladesh; this erosion-induced widening of the river system increases its capacity to transport massive sediment down steam [23]. Altogether, these rivers increase the country’s susceptibility to modifications in fluvial forms driven by extreme weather, which influences people, culture, the economy, and the environment [24]. Conversely, during the dry winter months (November-February), due to upstream control and water withdrawal, the GBM river system’s decreased flow has had significant socioeconomic and environmental consequences on riparian Bangladesh, including the southwest coastal riparian region. In addition, the Bangladeshi coasts are experiencing sedimentation in the southwestern areas, the loss of freshwater, high salinity intrusion, and riverbank erosion due to impediments like barrages and dams over transboundary watercourses [21]. Table 1 illustrates the disasters related to the river morphology and their subsequent impact on the riparian environment of coastal Bangladesh.

**Table 1:** The influences of fluvial morphology related to natural disasters in the riparian environment in southwest coastal Bangladesh (Adapted from [24]).

Hazards type	Impact on the riparian environment	Source
Flooding	Sedimentation, erosion, channel shifting, change in water discharge, debris flow, inundation, loss of agricultural production, and biodiversity destruction.	[24, 25]
Riverbank erosion	Bank failure, land property loss, deterioration of ecosystem, wide river valley, loss of vegetation, and supplies source material for char formation.	[24]
Channel Shifting	Riverbed rise, increasing flood, sand, and silt deposition vulnerability in agricultural land, river island (char) development, and multithreaded river channels.	[24]
Sedimentation	Fluvial landscape formation, inaccessibility, abandoned channels, and unavailable water for irrigation, nature-based income source.	[24]

Drawing on literature, the following have emerged as the critical characteristics of the socio-ecological system of the coastal riparian region in Bangladesh.

1. In tandem with climatic disasters, few non-climatic drivers cause massive damage to the socio-ecological system of the riparian region in the southwest coastal part of Bangladesh [26].
2. High input-driven agricultural operations, fodder production, livestock rearing, and shrimp culture upstream release a vast amount of nutrient-rich substances into coastal riparian floodplains system in Bangladesh, which degrade the soil and water environment [27].
3. Several other activities such as building embankments, dykes, polders, shrimp enclosure, water extraction for irrigated agriculture, and residential and commercial use also affect riparian environments in the entire southwest coastal riparian region in Bangladesh [28].
4. Likewise, tidal river dredging, channeling, modification for navigation, tourism, and urban development has enhanced the severity of climatic impacts on the livelihood, health, nutrition, food security, and well-being of the riparian communities [26].

Bangladesh is a country particularly vulnerable to climate change and one which is strongly affected by its impacts. Several works emphasise the value of and the need for local adaptation measures [e.g., 29–31]; therefore, this paper is timely since it fosters the knowledge on adaptation at the local level for riparian households and identifies the barriers to adaptation.

We present a case study from the Shyamnagar Upazila, a highly vulnerable coastal area in Bangladesh’s southwest region (SWCR). The main research question addressed is "to what extent does climate change affect riparian populations in Bangladesh and which actions are being taken towards adaptation?”

This article proceeds as follows. In the next section, we describe how climate change affects the riparian settlements in southwest Bangladesh. We then present our methodology, focusing on the study area and population, sampling, questionnaire, and data collection, and analysis. This section is followed by the results and discussion section, which focuses on the exposure of populations living in the riparian zone at the subdistrict level, household vulnerabilities, adaptation strategies, and the barriers to adaptation. Finally, we conclude with suggestions for possible policy measures from the study’s observations.

## 2. Climate change and riparian settlements in the southwest Bangladesh

Bangladesh’s population is 166 million [32] in South Asia, of which about 30% are coastal inhabitants [33], half exposed to coastal hazards. For being a lower riparian county of the Ganges-Brahmaputra river system that flows from the Himalayan range to the Bay of Bengal (BoB), an enormous volume of the water passes through the Ganges-Brahmaputra-Meghna (GBM) low-lying deltaic plain before discharging into the BoB (Fig 1).

Bangladesh’s climate vulnerability is characterised by very high physical exposure due to being a low-lying, lower-riparian nation often convulsed by destructive floods [34], sensitivity to climate-related risks, and a low adaptive capacity to cope and adapt to them. The high exposure is reflected in the Climate Risk Index [35], which places Bangladesh as the 7th most affected country by extreme weather events from 2000–2019. In addition, low adaptive capacity is often linked to overpopulation, abject poverty, and low socioeconomic development country status (LDC) [33, 36–39]. Bimal Kanti and Harun [40] highlighted the impacts of tropical cyclones and storm surges under sea-level rise, particularly on the coastal flooding and saltwater intrusion on fresh water and the surrounding soil, in their book focused on the vulnerability and adaptation of Bangladesh’s coastal region.

Several authors have primarily related the high climatic exposure and sensitivity of the coastal regions of Bangladesh [e.g., 27, 38, 41–45] to some of the following factors:

1. The dominance of floodplains and a low-elevation coastal zone: about half of Bangladesh is just 5 meters above the mean sea level (MSL).
2. A massive volume of the Himalayan glaciers’ melted water passes over this low-lying deltaic plain before discharging into the BoB.
3. Extreme weather events (EWEs), especially sea floodings and seawater intrusion.
4. The GBM system is essential for the natural resource-dependent population.

Severe flooding, catastrophic cyclone and storm surges, increased salinity and drought, reduced agricultural production, a lack of safe drinking water, and waterlogging because of rising sea levels are some of the effects of climate change in the coastal region [25]. Bangladesh is considered the country most susceptible to tropical cyclones worldwide. Devastating tropical cyclones have hit the deltaic nation’s coastal regions, leaving mess, destruction, and misery behind [25]. It is also considered the third most vulnerable to sea-level rise regarding the number of people affected [46].

Scientific research has already demonstrated the extent of saline and brackish water bodies due to rising sea levels in the global coastal regions, including Bangladesh [47]. For example, river salinity in the southern districts of Patuakhali, Pirojpur, Barguna, Bagerhat, Khulna, and Satkhira has risen by 45% since 1948 [47]. Globally, agriculture, food security, and human health are seriously impacted by salinity intrusion and the increasing salinity in water and soil. It also contributes to the chronic malnutrition and poor calorie intake of coastal populations in emerging and underdeveloped nations like Bangladesh [47].

According to Golder et al. [48] and Rahaman and Rahman [25], Bangladesh’s economy primarily depends on agriculture. Consequently, a sizable fraction of the population is employed directly or indirectly in agro-based activities [25]. Declining crop yield and precarious food security are consequences of global warming. Extreme temperatures, changing precipitation patterns, droughts, floods, waterlogging, and saline intrusion have severely impacted Bangladesh’s agricultural production [25]. Due to their extensive reliance on agriculture, people in low-income developing nations like Bangladesh are particularly vulnerable to the climate crisis. According to emerging data from coastal Bangladesh, males are increasingly leaving their homes in coastal districts or are at least inclined to move to surrounding urban centres or the nation’s capital to support their families [49].

Floods and storms are the most frequent EWEs affecting coastal areas [38, 50]. For instance, families living in the low-lying areas (e.g. natural drainage/depression) of the RZs are regularly exposed to fluvial and sea flooding. These marginalised peoples are often the landless class occupying the *‘Khas’* land (land owned by the Government of Bangladesh). These coastal riparian communities adapted to flooding during monsoon (June-July) and post-monsoon periods (August-September). However, because the riverbed rises due to heavy siltation, which overflows the natural levee/embankment/polder, causing severe coastal flooding [27, 51, 52]; some previous works on climate change and riparian communities in Bangladesh are placed on Table 2, which also describes their scopes.

**Table 2:** Some studies on climate change in river areas in Bangladesh.

Study	Results	Reference
Climate change adaptation through local knowledge in the northeastern region of Bangladesh.	Identified adaptations strategies used by communities in selected areas in Bangladesh	[53]
Riverbank erosion, population migration, and rural vulnerability in Bangladesh.	Presents a correlation between river bank erosion and migration in the Kazipur Upazila, at Sirajgonj District	[54]
Autonomous adaptation to riverine flooding in Satkhira District, Bangladesh.	Listed various autonomous adaptation practices and considers their implications for adaptation planning	[55]
The Costs of Living with Floods in the Jamuna Floodplain in Bangladesh	Identified the economic costs of changes in livelihood conditions over recent decades in a large floodplain area in northwest Bangladesh, with lessons learned on how to handle floods	[56]
Vulnerability to climatic change in riparian char and riverbank households in Bangladesh: Implication for policy, livelihoods and social development	Identified the vulnerabilities in livelihoods at the community level and proposes measures to address climate-driven riverbank erosion, which causes the loss of land and impacts riparian households.	[22]
Adaptation to river erosion-induced displacement in Koyra Upazilla of Bangladesh.	Describes a set of adaptation options at the Koyra Upazila and suggests means to optimise them	[57]

Source: The authors based on various sources

Seawater intrusion during high tide and stormy weather in the BoB are widespread throughout the dry winter (November-February) and the onset of rainy summer (April-May), exposing the coastal communities. During this period, the *‘Gheer’* owners (shrimp cultivators) forcefully keep the sluice gates open to bring saltwater inside the polders; this creates conflicts with the rice/crop growers. Salinity also builds up due to natural processes throughout the winter (November-February) and starts declining during the onset of monsoon (April-May) [52, 58–60].

However, during the entire period of wet season rice (*Aman* rice planted in May-June and harvested in November-December) cultivation, the riparian community feels better off as there are employment and earning opportunities. Moreover, many leave to pursue freshwater and marine fishing in the nearshore, offshore rivers, and estuaries in and around SMF. These activities often create localised shortages of male farm labourers, which provide the opportunity for the female to earn money. Usually, casual labourers maximise their earnings in the wet season’s farming to offset the dry winter income loss as most land parcels remain fallow [23, 58, 60].

The projected climate scenarios for 2050 will likely increase the exposed area by 14 percent to 70 percent due to a sea-level rise (SLR) of 27 cm and a 10 percent intensification of wind speed, given a +1 and +3 meter inundation depth, respectively [61]. About 33 percent of Bangladesh is expected to experience regular flooding under an SLR of 62 cm by ≈2080. Another 16 percent of the land is likely to become waterlogged if rainfall synchronises with such SLR levels [27, 62, 63]. Under an SLR of about 0.88 m by ≈2100, most low-lying non-embanked coastal areas may be completely inundated [64].

The southwestern coastal region of Bangladesh (SWCR) overlaps riparian zones of many tidal rivers such as Rupsha, Pasur, Shibsa, Ichamoti-Jamuna, Kholpatua-Arpangachia, Bhola-Baleswar that ultimately drain to the BoB. The low-lying SWCR is historically vulnerable to various EWEs (cyclonic surges, drought) and their associated disasters (e.g. sea flooding, salinisation, river erosion). The SWCR might experience even more challenges as most of it is just one meter above MSL [38, 60, 65].

The rivers flowing into the BoB drain over the Sundarbans mangrove forest (SMF) that covers a large part of 6,000 km^2^ at the outside limit of the deltaic plain region. The impact zone of Sundarbans is the riparian region of the three major rivers, Baleswar, Shibsa, and Pasur [66]. The freshwater flowing from the upper catchment through the riparian zones to the tidally influenced SMF is essential for the ecosystem’s health; it pushes the saltwater flow back from the sea [27, 59, 66].

The SMF provides a significant first line of defense against extreme weather events, while the human-made earthen embankments along the nearby rivers provide the second barrier. Nonetheless, the inhabitants of these three rivers’ riparian communities are still at risk of climatic events. Climate scenarios project an SLR of 0.8 m and more frequent extreme events by the last part of this century [42, 67–71].

A 10 km wide buffer zone called the ecologically critical area (ECA) surrounds the Sundarbans Protected Area (SPA). Specific development activities are controlled in the SPA, but human habitation is not limited. This intrusion of humans and their activities (forest clearance for saline shrimp farming) in the riparian zone’s forested land affects the ecosystem by altering the salinity levels. A well-balanced salinity provides a habitat for the rich biodiversity of flora and fauna, breeding grounds, and nurseries for vertebrate species [59, 66, 72].

The actual impact zone of the Sundarbans covers around 50 km from the edge of the SPA. The three significant rivers’ riparian communities live in the impact zone of the Sundarbans ecosystem [59]. However, these communities’ vulnerability and livelihood differ in many respects. For example, they live in different salinity ranges (onset of the dry season), which can be characterised as follows: [59, 60, 66, 73].

- <2 ppt (oligohaline zone) in Sarankhola Upazila along the Baleswar river (the eastern edge of the Sundarbans). People have engaged primarily in freshwater-dependent livelihoods; however, peak dry season salinity could rise to 5 ppt.
- 2–4 ppt (mesohaline zone) in MonglaUpazila along the Pasur River (northern edge of the Sundarbans). People are engaged in freshwater, crop agriculture, and saltwater aquaculture; however, peak dry season salinity could rise 5-10ppt.
- >4 ppt (polyhaline zone) in Shymnagar Upazila along the Kobadak-Shibsa River (on the northwest edge of the Sundarbans). People are engaged in primarily saltwater-dependent livelihoods; however, peak dry season salinity could typically rise 10-15ppt.

Among the climate-vulnerable riparian settlements in Shyamnagar Upazilla (subdistrict) in the Satkhira district, the riparian communities in Munshiganj Union Parishad (the lowest tier local government unit) were purposely chosen as the study sites. This is because these communities have a higher incidence of poverty as climatic and non-climatic factors threaten their livelihoods [74, 75]. Also, a higher proportion of the population and various occupational groups dependent on SMF’s resources live in a marginalised pocket near the Sundarbans impact zone, close to the India-Bangladesh border. A general explanation of the riparian study sites [27, 76] is given later in this article.

As a case study, the riparian settlements between the tidal rivers Ichamoti-Jamuna and Kholpatua-Kobadak (a tributary of the mighty Shibsa River) are presented in the following sections.

## 3. Methodology

### 3.1 Study area and population

The paper uses a case study approach to analyse the climate-related impacts on settlements in Bangladesh’s SWCR, the Shyamnagar Upazilla, and the Satkhira district. The studied area is 1,968 sq. km, whereas the Sundarbans Reserve Forest (SRF) covers 1,535 sq. km, of which only 429 sq. km are habitable areas [77]. The SRF is one of South Asia’s most vulnerable geographical locations due to the long-term climate-related impacts on this highly exposed coastal area. The studied area is adjacent to the Sundarbans mangrove forest (SMF) world heritage site, 60 km north of the BoB, located east of the South 24 Pargana district of West Bengal in India. Survey data was collected from three villages of Munshiganj Union Parishad: Kachukhal, Kultali, and Mothurapur. The study sites are located in the riparian region of the transboundary river (between India and Bangladesh), Ichamati-Jamuna (to the west), and the river Kholpatua (to the east).

### 3.2 Selection of the sample, survey form, and data gathering

A list of households in the three studied villages was first collected from the Department of Agricultural Extension (DAE). Then, village resident data were collected by local-level DAE officials. The study villages consist of 269 riverine households. The number of people in the villages studied and their households were relatively small. As the population is known and finite (269), the sampling size was determined by Yamane’s formula (1967) [78].


$$n=\frac{N}{1+Ne^{2}}$$

Where, n = sample size, N = population, e = error margin (in %)

As the population is more homogenous in terms of their higher dependency on climatically affected, natural resource-dependent livelihoods, the authors believed that choosing an error margin of 10 percent would significantly reduce the sample size and survey costs without significantly compromising the data quality. However, taking 5% is more common in a heterogeneous setting. As the population is also more homogenous in terms of their exposure to multiple disasters, the authors believed that the sample size, despite being small, could significantly represent the population. Therefore, the authors allowed a 10 percent margin of error and surveyed 73 households selected randomly from 269 families (≈27% of total households). The survey evaluation unit was households, and data were collected from the household heads (either male or female). The interviewees were selected using the random sampling technique across all settlements. In case of a non-response, which occurred in less than 1 percent of the actual sample, the enumerators went on to examine the following family in search of reaching the needed number of respondents in each village. A Focus group discussion (FGD) took place in all surveyed villages in the presence of 10 to 12 heads of households to attain their opinions to cross-validate the details acquired from the inquiries. The authors created a semi-structured survey questionnaire to gather information through in-person interviews in April-May 2019. Two trained enumerators carried out the survey. The questionnaire contains data on households’ vulnerability, response strategies, and barriers to adaptation.

### 3.3 Methods for data analysis

A Severity Index of Vulnerability (SIV) was developed to calculate the strength of the respondent’s opinions through a 5-point Likert Scale ranging from Very Low to Very High [79, 80]. The respondents were asked to scale the various climatic events they often experienced and the scale’s corresponding value, ranging from 1 (Very Low) to 5 (Very high). The SVI was made in the following manner:

$$SVI=\frac{\sum_{i=1}^{5}X_{i}\;F_{i}}{N}$$

Xi = Scale rate taking the precedence of the climatic event, Fi = Frequency of responses, N = number of interviewees, and i = 1, 2, —5. The SVI value ranges from 1: very low vulnerability to 5: very high vulnerability. The interviewees also answered about their adaptation strategies and adaptation usages identified through literature reviews and FGDs.

### 3.4 Inclusivity in global research

Additional information regarding the ethical, cultural, and scientific considerations specific to inclusivity in global research is included in the S1 File.

## 4. Results and discussion

The study investigates the riparian population’s exposure, vulnerability, and adaptation to climate-related risks. The results will, therefore, focus on these three main components:

- Household exposure and vulnerabilities;
- The adaptation strategies being deployed and;
- The barriers to adaptation

Due to their importance, these components are described and discussed in the following sections. These outcomes help advance our understanding of the vulnerability and adaptation practices of the riparian people of coastal Bangladesh. Hydro-geomorphologically, as this coastal riparian region is different from the surrounding coastal flood plains, the vulnerability and adaptation practices are different. In the surrounding coastal flood plains, the riparian zones are not easily identifiable because massive precipitation in the wet season (rainfall of about 1800 mm) allows the growth of tall trees and shrubs along the river corridor and the nearby flood plains. However, in the studied riparian region, riparian characteristics are easily identifiable in the dry winter. During this season, the river’s additional water allows large trees and shrubs to grow along the river bank. However, the salt-affected agricultural field away from the river does not get enough water to grow large trees and shrubs. Therefore, the agricultural field remains fallow due to salt’s high deposition in topsoil and moisture stress.

### 4.1 Household exposure and vulnerabilities

Households in the study area are accustomed to being exposed to several climatic hazards every year [58]. The participants responded about their perception of climate-related vulnerability and impact. SVI was used for understanding vulnerability. Salinity is ranked as the primary source of vulnerability, followed by an increase in temperature (4.32), riverbank erosion (3.38), and flood (3.22) (Table 3). Table 3 also presents the percentage of the population severely affected by various extreme climatic events. Salinity is ranked number one primarily because salinity continues to increase from October to March. In March-April, salinity in topsoil goes as high as 20 dS/m due to the capillary rise of saline groundwater.

**Table 3:** Household vulnerabilities to climate change and extreme weather events.

Climatic events (N)	Level of vulnerability (response in %)			Severity Score (SS)	Rank of SS
	Very High/High	Medium	Low/Very low		
Salinity (N = 71)	100%	0%	0%	4.90	1
Increase in temperature (N = 71)	97.2%	2.8%	0%	4.32	2
Riverbank erosion (N = 61)	63.9%	34.4%	1.6%	3.38	3
Flood (N = 61)	65.6%	29.5%	4.9%	3.22	4
Drought (N = 66)	36.3%	56.1%	7.6%	3.20	5
Sea-level rise (N = 62)	37.1%	59.7%	3.2%	2.97	6
Rainfall (N = 65)	27.9%	56.9%	15.4%	2.93	7
Cyclones and Storm surge (N = 64)	43.8%	48.4%	7.9%	2.90	8

Source: the authors

Moreover, after each episode of cyclonic surges very thick salt layer remains on the topsoil, making it unproductive for years. As the study riparian zone, due to its closeness to the cyclonic path, experience recurrent exposure to the cyclone of varying intensities that carry giant seawater from the sea (Bay of Bengal), the inhabitants of the riparian zone badly suffer. Even the whole riparian ecosystem (both plant species and small animals) gets disturbed; only salt-tolerant species survive. Salinity is, therefore, harmful not only for the crop, horticulture, and (animal) fodder production but also for livelihood, as the availability of drinking water becomes scarce in the entire dry season. The riparian settlements have long suffered from salt accumulation on agricultural fields and aquaculture enclosures even after five years of super cyclone Aila devastated Bangladesh’s coastal zone in 2009. The rainfall could not wash out the accumulated salt layer from agricultural fields for years. Due to riparian landforms, rainwater quickly drained to rivers before washing out the salt layer. Harvest loss/failure was a recurrent event; therefore, riparian settlements’ economic suffering and hardship were more than communities living in other flood plains in the coastal tract.

The studied riparian zone is just along the rivers that make way for cyclonic or wave surges to travel further inland (along the river corridor) from the BoB. Therefore, compared to the floodplain community located away from rivers, the riparian settlement experiences more recurrent coastal flooding events in the wet season (June-September). This localised flooding situation in the riparian regions is often getting complicated as the indiscriminate conversion of rice fields to shrimp enclosures restricts natural/gravity flows of draining water. Thus, trapped water creates a submersed condition for a prolonged period. Moreover, due to the river channel’s proximity, the riparian settlements experience frequent riverbank erosion occurrences as wave action erodes the riverbank’s inner side. This erosion results in frequent shifting/relocation of landing port/landing station of water vessels, which seriously affects their mobility as water transport is their primary mode of mobility.

On the other hand, due to the quick passing of water overland flow/surface runoff in the wet/rainy season (June-September) to nearby steam channels/rivers, the riparian community experiences sheet and gully erosion. Both these types of erosion result in loss of soil nutrients (from topsoil) in the study riparian settlement, which renders low productivity of wet season crops. As the entire study riparian region depends mainly on wet season rice (due to high salinity, the land remains fallow in winter), lowering productivity puts an additional layer on their already threatened food and nutritional security.

However, the impacts of climate induced-events in riparian areas are felt differently by different social groups [22, 58]. Impacts of coastal flooding and heavy rainfall are felt more by peasant farmers, aquaculturists, subsistence fisheries, petty traders, and rural transport workers [58, 76, 81]. The increased salinity is felt more by peasant farmers and other on-farm occupation groups [82]. Due to salinity intrusion, many lands are unsuitable for crop cultivation, affecting small and landless farmers due to their higher livelihood dependency on crop cultivation. Saline land is now brought under shrimp cultivation but is dominated by affluent farmers [83]. By contrast, the impacts of cyclonic surges are felt more by people engaged in both subsistence and commercial fishing in the rivers, as well as nearshore and offshore areas, because cyclonic events impede the entire fish value chain by affecting catching, preservation, processing, transportation, and marketing of fish products [58, 76, 81]. Wage labourers severely suffer from disaster because they have limited ways to support themselves, and their physical labour cannot be capitalised on during disaster periods [27, 58, 84]. Many farmers also experienced the loss of cropland due to riverbank erosion problems, which increased their vulnerability. Due to the river’s proximity, riparian households are more prone to fluvial and pluvial flooding [36].

### 4.2 Adaptation strategies used

**Fig 2:**
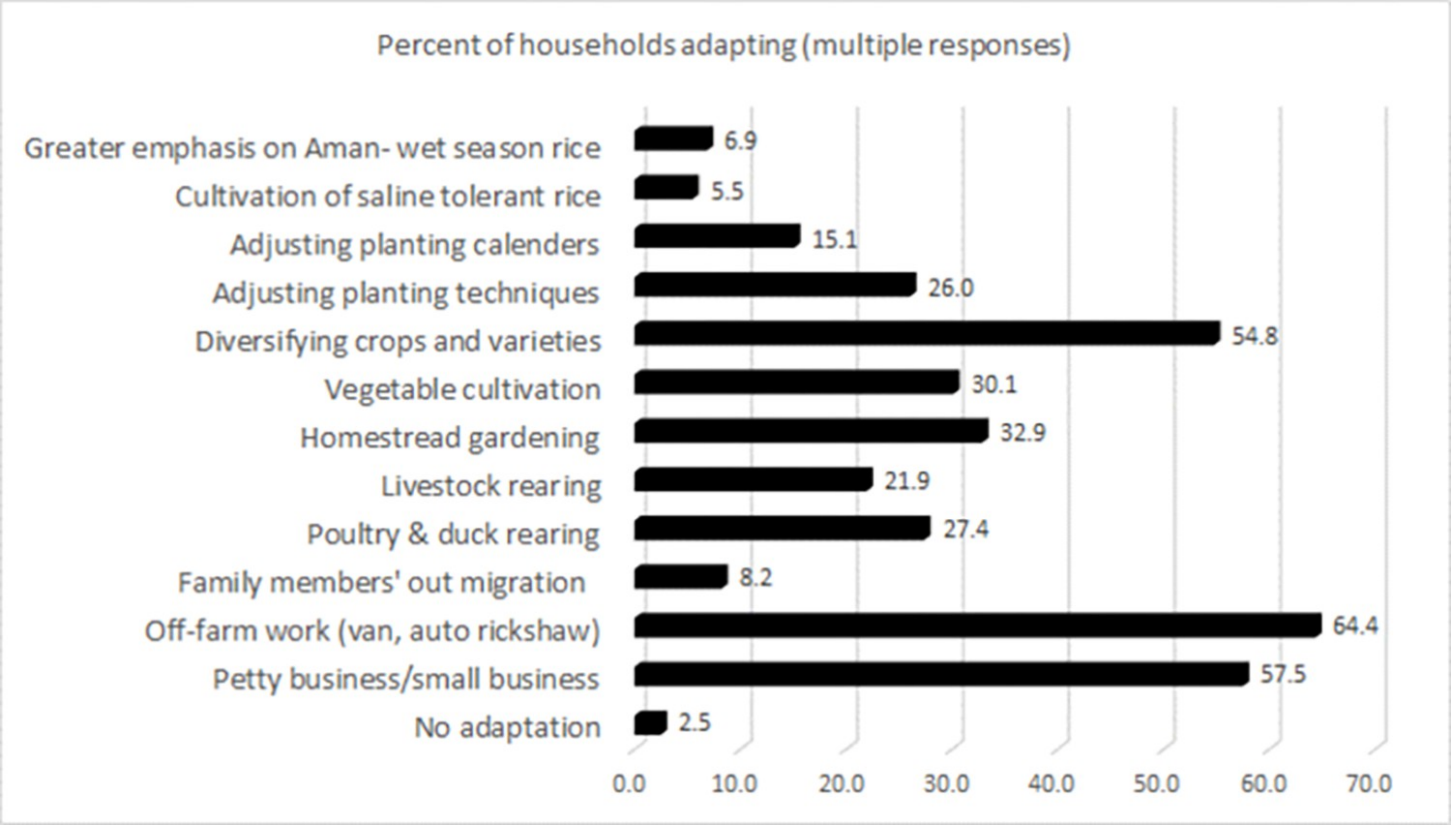
Adaptation strategies used by households. Source: the authors.

Climate adaptation is a type of adjustment in biophysical, social, and institutional systems to minimise the impacts of climatic events on the environment and livelihoods [22]. However, adaptation strategies are often place-based, dynamic, and even value-driven and can change over time [85]. This study has identified about a dozen adaptation strategies employed by the riparian people (Fig 2).

Most of the households in the riparian settlements have adopted specific adaptation strategies. For example, they may have engaged in livestock rearing, homestead grading, or vegetable cultivation combined with small poultry and duck rearing businesses. Fig 2 demonstrates that most farmers adopted off-farm work (64.4 percent), followed by creating small businesses (57.5 percent), which can be considered a non-agriculture adaptation. The most common agricultural adaptation strategies were diversification of crops/cultivars (54.8 percent), backyard gardening (32.9 percent), and vegetable production (30.1 percent). Most adaptation strategies can be considered individual-level adaptations, most notably the non-agricultural measures [58, 76, 81, 84].

Table 4 summarises the most favoured adaptation strategies of the most vulnerable groups. This may inform targeted interventions to promote adaptation among riparian communities.

**Table 4:** Vulnerability-specific adaptation measures used by impacted families.

Families highly impacted by (N)	Common adaptation strategy (response in %)		
	Most frequent	2^nd^ most frequent	3^rd^ most frequent
Salinity (N = 71)	Petty trade/income diversification (73.24%)	Crop diversification (63.38%)	Homestead gardening (40.84%)
Increase in temperature (N = 71)	Petty trade/income diversification (70.42%)	Crop diversification (63.38%)	Homestead gardening (39.44%)
Riverbank erosion (N = 39)	Petty trade/income diversification (76.92%)	Crop diversification (61.54%)	Homestead gardening (48.72%)
Flood (N = 40)	Petty trade/income diversification (72.5%)	Crop diversification (55.00%)	Homestead gardening (50.00%)
Drought (N = 24)	Petty trade/income diversification (70.83%)	Crop diversification (54.17%)	Homestead gardening (54.17%)
Sea-level rise (N = 23)	Homestead gardening (69.56%)	Petty trade/income diversification (60.87%)	Vegetable cultivation (52.17%)
Rainfall (N = 18)	Petty trade/income diversification (66.67%)	Crop diversification (61.11%)	Homestead gardening (50.0%)
Cyclones and Storm surge (N = 28)	Petty trade/income diversification (67.86%)	Crop diversification (64.28%)	Off-farm work (46.43%)

Note: N indicates the number of respondents

Source: the authors

Unfortunately, neither government agencies nor grassroots organisations, including local NGOs, provide enough support in credits, agricultural inputs, training, and marketing infrastructure development to promote adaptation.

Farmers with small or no cultivable land adopted non-agricultural adaptation practices mostly. They mentioned that their scope of doing off-farm work (van, auto-rickshaw driving) has increased due to improved road communication. Many farmers were also involved with small businesses; nevertheless, they did not receive adequate support from the government and NGOs to become entrepreneurs. Farmers opined that increased adoption of backyard gardening and poultry/duck rearing helped them improve their livelihoods. These activities have been intensified due to the lower reliance on fisheries. Therefore, they can be regarded as a modality of adaptation to changing conditions.

### 4.3 Barriers to adaptation

**Fig 3:**
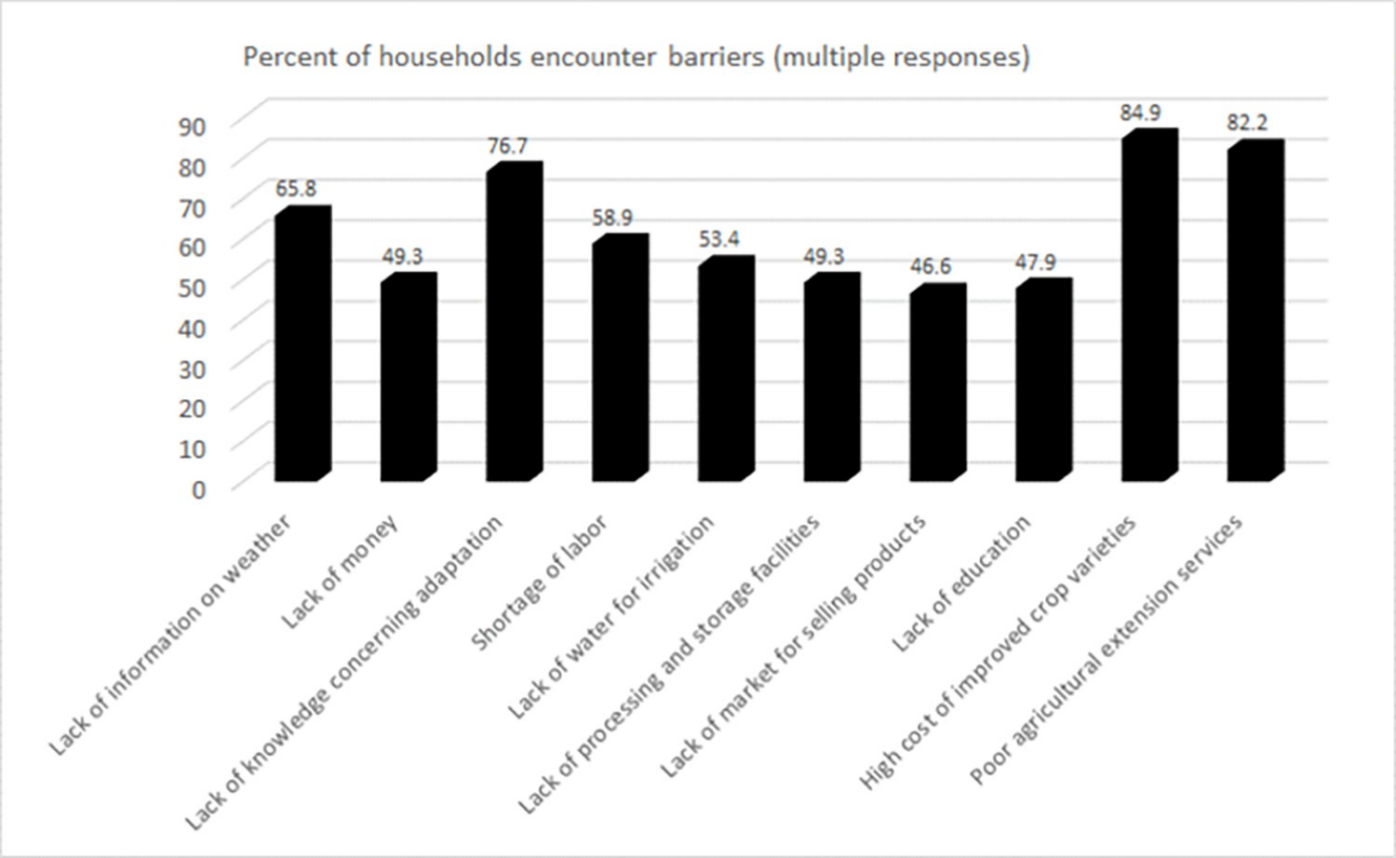
Barriers to adaptation. Source: the authors.

Respondents were also asked to list their perceived barriers to adaptation to climatic impacts. While they were found to adopt various adaptation strategies, they also mentioned some barriers to adaptation. As seen in Fig 3, the main barriers to adaptation were the high cost of improved varieties (84.9 percent), inadequate agricultural extension services (82.2 percent), and a lack of knowledge of adaptation (76.7 percent).

They also mentioned a lack of capital, market facilities, and education as a barrier to adaptation.

Lack of access to institutional credit, modern agricultural inputs, and implements limits their ability to adapt [23, 60]. Respondents also mentioned that their limited or no access to updated (and real-time) climate and weather services is also a barrier to adaptation. This finding is consistent with some other studies [e.g. 76, 86] that have identified a lack of information on weather and climate as a barrier to adaptation in varied contexts. Knowledge about these barriers is relevant because it shows that apart from economic support for investments to reduce vulnerability, providing information and advice to local communities is an essential element that should not be overlooked.

Table 5 summarises the most favoured adaptation-specific barriers. This could inform targeted interventions to build resilience among riparian communities.

**Table 5:** Adaptation-specific barriers.

Most favoured adaptation among the most vulnerable (N)	Common barriers to adaptation (response in %)		
	Most frequent	2^nd^ most frequent	3^rd^ most frequent
Petty trade/income diversification (N = 40)	High cost of improved agriculture (70.0%)	Lack of education (62.50%)	Lack of adaptation knowledge (60.0%)
Crop diversification (N = 39)	Lack of adaptation knowledge (79.49%)	High cost of improved agriculture (79.49%)	Lack of water for irrigation (86.92%)
Homestead gardening (N = 29)	Shortage of labour (75.86%)	Lack of irrigation (72.41%)	Lack of money to invest (72.41%)
Vegetable cultivation (N = 28)	Lack of water for irrigation (78.57%)	Shortage of labour (64.28%)	Lack of money to invest (60.71%)
Off-farm work (N = 24)	High cost of improved agriculture (75.0%)	Lack of adaptation knowledge (62.5%)	Lack of education (54.17%)

Note: N indicates the number of respondents

Source: the authors

The research performed has some limitations. Firstly, the sample cannot be regarded as representative of the whole population of the Sundarbans since the authors encountered difficulties in collecting data and therefore have adjusted the methods accordingly. Secondly, although interviews were held with local people and in the local language, it is still possible that some respondents–from a population that is very poor and deprived of education opportunities–did not fully understand the nature of the research and did not adequately respond to the matters raised during the interviews. Finally, the interviews were focused mainly on aspects of climate-related vulnerability and impacts, so there may also be issues and concerns which were not fully captured in the study. Despite these shortcomings, this study makes a noble contribution to the literature on the adaptation behaviour of the riparian community against the climate-related risks and vulnerabilities in Bangladesh’s coastal landscape, especially in the Sunderbans’ impact zone.

### 4.4 Policy implications

The evidence from the study suggests that government policies are needed, which may assist riparian communities in tackling the problems they face. Some measures which should be taken into account include:

- The provisions of saline-resistant crops to local farmers may replace the current ones, which are unsuitable for water growth with high salinity.
- The development of infrastructure projects to increase the availability of water for drinking purposes and also for agriculture (e.g. rainwater harvesting).
- A boosting of agricultural extension services to help those who wish to remain in farming to obtain better yields.
- Encouraging diversification of income-generating activities to reduce the reliance on agriculture.

In addition, a long-term support programme is needed, possibly funded by international donors, to assist riparian settlements in implementing several other measures, such as maintaining functional hydrologic regimes in local watersheds- which may help to increase their adaptive capacity.

## 5. Conclusions

This paper reports on a study aimed at providing an overview of the impacts of climate change on riparian communities in Bangladesh. Consisting of an empirical study involving a sample of riparian communities in Bangladesh, this paper provides additional evidence on how climate change affects livelihoods and the dynamics of communities in Bangladesh, which is one of most vulnerable countries as far as climate change is concerned.

This paper has some limitations, one of which is that it focused on the Sundarbans, not other areas in Bangladesh. Also, the survey engaged a small group of inhabitants of riparian communities- three villages consisting of 269 riverine households- and cannot be regarded as representative of the whole area. Finally, the study focused on climate-related risks and did not dwell extensively on socioeconomic components. Despite these constraints, this study provides a welcome addition to the literature since it presents an overview of the extent to which climate change impacts riverine communities in the Sundarbans and describes the extent of the problems they are exposed to.

This paper has four main implications. Firstly, the data gathered suggest that seawater intrusion and the increase in salinity are significantly affecting local communities. This is because they interfere with the physiology of fauna and flora. The high emphasis on salinity is associated with its detrimental impact on crop production and has substantial economic consequences. Likewise, salinity affects health due to the limited availability of drinking water. The importance of salinity in this area is a striking difference compared to other riparian settlements elsewhere in the world, which can rely on normal river water.

Secondly, floods and increased riverbank erosion are also additional pressures local communities face.

A further implication of this article is that it has shown that climate-related impacts do not homogenously negatively affect riparian communities and settlements. For instance, coastal flooding predominantly affects those who depend on agriculture, aquaculture, or subsistence fisheries, whereas increases in salinity most affect those engaged in farming activities.

Finally, the research has shown that riparian communities implement diverse strategies to adapt to the various climate conditions affecting their livelihoods. Such strategies include a mix of income-generating activities (e.g. a combination of livestock rearing and homestead grading), off-farm work, and small business creation to reduce their dependence on agriculture.

As far as the barriers to adaptation are concerned, it is clear that the high costs of improved varieties of crops (e.g. salt-resistant ones) and inadequate agricultural extension services represent critical limiting factors, along with the limited knowledge of appropriate adaptation strategies.

Future research may focus on the impacts of climate change on health care, which has been severely affected by the COVID-19 pandemic. Further studies may also be focusing on producing a list of various responses seen across different communities in Bangladesh. Moreover, it could be useful to undertake research on how minority groups are being affected by climate change, identifying measures to reduce their vulnerability and increase their resilience.

Moving forward, more efforts to restore mangrove ecosystems are also needed. Whereas mangrove restoration cannot fully protect riparian settlements from the impacts of a changing climate, it may help to increase their resilience and, among other things, make the local settlements less physically and economically exposed to extreme events.

## Supporting information

S1 File Questionnaire on inclusivity. (DOCX)

S2 File Supporting data. (PDF)

## References

[1] Zaimes GN, Iakovoglou V, Emmanouloudis D, Gounaridis D. Riparian areas of Greece: their definition and characteristics. J Eng Sci Tech Rev. 2010;3(1): 176–83.

[2] González E, Felipe-Lucia MR, Bourgeois B, Boz B, Nilsson C, Palmer G, et al. Integrative conservation of riparian zones. Biol Conserv. 2017;211: 20–9.

[3] Nóbrega RL, Ziembowicz T, Torres GN, Guzha AC, Amorim RS, Cardoso D, et al. Ecosystem services of a functionally diverse riparian zone in the Amazon–Cerrado agricultural frontier. Glob Ecol Conserv. 2020;21: e00819.

[4] Sabo JL, Sponseller R, Dixon M, Gade K, Harms T, Heffernan J, et al. Riparian zones increase regional species richness by harboring different, not more, species. Ecology. 2005;86(1): 5662.

[5] Ffolliott PF, DeBano LF. Riparian areas of the southwestern United States: hydrology, ecology, and management. Boca Raton, Florida: CRC Press; 2003.

[6] Naiman RJ, Bechtold JS, Drake DC, Latterell JJ, O’Keefe TC, Balian EV. Origins, patterns, and importance of heterogeneity in riparian systems. In: Lovett GM, Turner MG, Jones CG, Weathers KC, editors. Ecosystem function in heterogeneous landscapes. New York: Springer; 2005. p. 279–309.

[7] Gurnell AM, Rinaldi M, Belletti B, Bizzi S, Blamauer B, Braca G, et al. A multi-scale hierarchical framework for developing understanding of river behaviour to support river management. Aquat Sci. 2016;78(1): 1–16.

[8] Naiman RJ, Decamps H. The ecology of interfaces: riparian zones. Annu Rev Ecol Syst. 1997;28(1): 621–58.

[9] Primack RB, Sher A. Introduction to conservation biology. Sinauer Associates, Incorporated, Publishers; 2016.

[10] Tonkin JD, Merrit DM, Olden JD, Reynolds LV, Lytle DA. Flow regime alteration degrades ecological networks in riparian ecosystems. Nat Ecol Evol. 2018;2(1): 86–93. doi: 10.1038/s41559-017-0379-0 29180707

[11] Zaimes G. Defining Arizona’s riparian areas and their importance to the landscape. In: Zaimes G, editor. Understanding Arizona’s Riparian Areas. The University of Arizona; 2007. pp. 1–13.

[12] Kumar D. River Ganges–historical, cultural and socioeconomic attributes. Aquat Ecosyst Health Manag. 2017;20(1–2): 8–20.

[13] Dimmitt MA. Fabaceae (legume family): Olneyatesota. In: Phillips SJ, Comus PW, editors. A natural history of the Sonoran Desert, Berkley and Los Ángeles, California. University of California Press; 2000. pp. 234–235.

[14] Ffolliott PF, DeBano LF, Baker MB Jr, Neary DG, Brooks KN. Chapter 4. Hydrology and impacts of disturbances on hydrologic function. In: Baker MB, Ffolliott PF, DeBano LF, Neary DG, editors. Riparian Areas of the Southwestern United States: hydrology, ecology, and management. New York: Lewis Publishers; 2003. pp. 51–76.

[15] Feld CK, Birk S, Bradley DC, Hering D, Kail J, Marzin A, et al. From natural to degraded rivers and back again: a test of restoration ecology theory and practice. Adv Ecol Res. 2011;44: 119–209.

[16] Mensing DM, Galatowitsch SM, Tester JR. Anthropogenic effects on the biodiversity of riparian wetlands of a northern temperate landscape. J Environ Manage. 1998;53(4): 349–377.

[17] Graf WL. Downstream hydrologic and geomorphic effects of large dams on American rivers. Geomorphology. 2006;79(3–4): 336–360.

[18] Hughes FM, Rood SB. Allocation of river flows for restoration of floodplain forest ecosystems: a review of approaches and their applicability in Europe. Environ Manage. 2003;32(1): 12–33. doi: 10.1007/s00267-003-2834-8 14703910

[19] Svejcar T. Riparian zones. 1. What are they and how do they work? Rangelands Archives. 1997;19(4): 4–7.

[20] Hunter ML Jr, Acuña V, Bauer DM, Bell KP, Calhoun AJ, Felipe-Lucia MR, et al. Conserving small natural features with large ecological roles: a synthetic overview. Biol Conserv. 2017;211(B): 88–95.

[21] Saha P, Ashraf A, Oyshi JT, Khanum R, Nishat A. A community-based approach to sustainable transboundary water resources management and governance in the South-West Coastal region of Bangladesh. Sustain Water Resour Manag. 2021;7(5): 1–13.

[22] Alam GMM, Alam K, Mushtaq S, Clarke ML. Vulnerability to climatic change in riparian char and riverbank households in Bangladesh: Implication for policy, livelihoods and social development. Ecol Indic. 2017;72: 23–32.

[23] Alam GMM, Alam K, Shahbaz M, Leal Filho W. How do climate change and associated hazards impact on the resilience of riparian rural communities in Bangladesh? Policy implication for livelihood development. Environ Sci Policy. 2018;84: 7–18.

[24] Sultana R, Paul SK. Exploring the Impacts of River Morphology Change Associated Natural Disasters on Teesta Riparian Environment of Bangladesh. In: Jana NC, Singh RB, editors. Climate, Environment and Disaster in Developing Countries. Springer Singapore; 2022. pp. 361–373.

[25] Rahaman MA, Rahman MM. Climate justice and food security: Experience from climate finance in Bangladesh. In: Walker T, Sprung-Much N, Goubran S, editors. Environmental policy: An economic perspective. John Wiley & Sons Ltd; 2020pp. 249–268.

[26] Haque MN, Rahman S, Saroar M, Morshed SR, Fattah MA. A geospatial approach for environmental risk susceptibility mapping of Khulna city in Bangladesh. Phys Chem Earth. 2022; Parts A/B/C: 103139.

[27] Saroar MM, Rahman MM, Bahauddin KM, Rahaman MA. Ecosystem-based adaptation: Opportunities and challenges in coastal Bangladesh. In: Huq S, Chow J, Fenton A, Stott C, Taub J, Wright H, editors. Confronting Climate Change in Bangladesh. Springer, Cham; 2019. pp. 51–63.

[28] Sresto MA, Siddika S, Haque M, Saroar M. Groundwater vulnerability assessment in Khulna district of Bangladesh by integrating fuzzy algorithm and DRASTIC (DRASTIC-L) model. Model Earth Syst Environ. 2021;6: 1–5.

[29] Parry ML, Canziani OF, Palutikof JP, van der Linden PJ, Hanson CE, editors. IPCC. Climate Change 2007: Impacts, Adaptation and Vulnerability, Working Group II Contribution to the Fourth Assessment Report of the Intergovernmental Panel on Climate Change. Cambridge University Press, Cambridge, United Kingdom; 2007.

[30] Pachauri RK, Meyer LA, editors. IPCC. AR5 Synthesis Report. Contribution of Working Groups I, II and III to the Fifth Assessment Report of the Intergovernmental Panel on Climate Change. IPCC, Geneva, Switzerland; 2014.

[31] IPCC. Summary for Policymakers. In Masson-Delmotte V, Zhai P, Pirani A, Connors SL, Péan C, Berger S, et al., editors. Climate Change 2021: The Physical Science Basis. Contribution of Working Group I to the Sixth Assessment Report of the Intergovernmental Panel on Climate Change. Cambridge University Press, Cambridge, United Kingdom and New York, NY, USA; 2021. pp. 3−32.

[32] BBS (Bangladesh Bureau of Statistics) Bangladesh Statistics 2020. Statistics and Informatics Division (SID) Ministry of Planning, Government of the People’s Republic of Bangladesh, Dhaka. 2020.

[33] Ahmad H. Bangladesh Coastal Zone Management Status and Future. J Coast Zone Manag. 2019;22(1).

[34] Hasan H. Manifestations of Climate-Induced Migration. Is South Asia ready to tackle this crisis? Academia Letters. 2021;1165.

[35] Eckstein D, Künzel V, Schäfer L. Briefing Paper. Global Climate Risk Index 2021. Who Suffers Most from Extreme Weather Events? Weather-Related Loss Events in 2019 and 2000–2019. Germanwatch e.V Office Bonn. 2021.

[36] Alam GMM, Alam K, Shahbaz M, Khatun MN, Leal Filho W. Strategies and barriers to the adaptation of hazard-prone rural households in Bangladesh. In: Leal Filho W, Nalau J, editors. Limits to Climate Change Adaptation. Springer International Publishing; 2019. pp. 11–24.

[37] Kreft S, Eckstein D, Melchior I. Global climate risk index 2017. Briefing Paper. Who Suffers Most From Extreme Weather Events? Weather-related Loss Events in 2015 and 1996 to 2015. Germanwatche.V. Office Bonn. Office Berlin; 2017.

[38] Leal Filho W, Modesto F, Nagy GJ, Saroar M, YannickToamukum N, Ha’apio M. Fostering coastal resilience to climate change vulnerability in Bangladesh, Brazil, Cameroon, and Uruguay: a cross-country comparison. J Mitig Adapt Strateg Gl Chang. 2018;23(4): 579–602.

[39] ND-Gain. ND-GAIN Country Index. University of Notre Dame [Internet]. 2018 [Cited 2018 November 20]. Available from: https://gain.nd.edu/our-work/country-index/

[40] Bimal Kanti P, Harun R. Climatic Hazards in Coastal Bangladesh. Non-Structural and Structural Solutions. Elsevier Inc.; 2016.

[41] DasGupta S, Das Dasgupta S. Globalisation and transnational surrogacy in India: outsourcing life. Lexington Books; 2014.

[42] Dastagir MR. Modeling recent climate change induced extreme events in Bangladesh: A review. Weather Clim Extrem. 2015;7: 49–60.

[43] MoFF’ Bangladesh Climate Change Strategy and Action Plan 2009. Ministry of Environment and Forests, Government of the People’s Republic of Bangladesh, Dhaka, Bangladesh; 2009.

[44] Nandy P, Ahammad R, Alam M, Islam A. Coastal ecosystem-based adaptation: Bangladesh experience. In: Shaw R, Mallick F, Islam A, editors. Climate Change Adaptation Actions in Bangladesh. Disaster Risk Reduction (Methods, Approaches and Practices). Tokyo: Springer; 2013. pp. 277–303.

[45] Rahaman MA, Rahman MM, Hossain MS. Climate-Resilient Agricultural Practices in Different Agro-ecological Zones of Bangladesh, In: Leal Filho W, editor. Handbook of Climate Change Resilience. Springer, Cham; 2020a. pp. 2337–363.

[46] Rahaman MA, Rahman MM, Rahman SH. Pathways of climate-resilient health systems in Bangladesh. In: Huq S, Chow J, Fenton A, Stott C, Taub J, Wright H, editors. Confronting Climate Change in Bangladesh. Springer, Cham; 2019. pp. 119–143.

[47] Rahaman MA, Rahman MM, Nazimuzzaman M. Impact of salinity on infectious disease outbreaks: experiences from the global coastal region. In Leal Filho W, Wall T, Azul AM, Brandli L, Gökcin Özuyar P, editors, Good Health and Well-Being Springer, Cham; 2020b. pp. 415–424.

[48] Golder P, Sastry R, Srinivas K. Research priorities in Bangladesh: analysis of crop production trends. SAARC J Agri. 201;11(1): 53–70.

[49] Ahmed S, Eklund E. Climate change impacts in coastal Bangladesh: Migration, gender, and environmental injustice. Asian Affairs. 2021;52(1): 155–174.

[50] Haq SMA, Ahmed KJ. Is Fertility Preference Related to Perception of the Risk of Child Mortality, Changes in Landholding, and Type of Family? A Comparative Study on Populations Vulnerable and not Vulnerable to Extreme Weather Events in Bangladesh. Popul Rev. 2019;58(2): 61–99.

[51] Mallick B, Ahmed B, Vogt J. Living with the Risks of Cyclone Disasters in the South-Western Coastal Region of Bangladesh. Environments. 2017;4(13).

[52] Wilson C, Goodbred S, Small C, Gilligan J, Sams S, Mallick B, et al. Widespread infilling of tidal channels and navigable waterways in the human-modified tidal delta plain of southwest Bangladesh. Elementa: Science of the Anthropocene. 2017;5:78.

[53] Anik SI, Khan MASA. Climate change adaptation through local knowledge in the north eastern region of Bangladesh. Mitig Adapt Strateg Glob Chang. 2012;17(8):879–96.

[54] Mollah T, Ferdaush J. Riverbank erosion, population migration and rural vulnerability in Bangladesh (a case study on Kazipur Upazila at Sirajgonj District). Environ Ecol Res. 2015;3(5):125–31.

[55] Fenton A, Paavola J, Tallontire A. Autonomous adaptation to riverine flooding in Satkhira District, Bangladesh: implications for adaptation planning. Reg Environ Change. 2017;17(8):2387–2396.

[56] Ferdous MR, Wesselink A, Brandimarte L, Slager K, Zwarteveen M, Di Baldassarre G. The Costs of Living with Floods in the Jamuna. Floodplain in Bangladesh. Water. 2019;11:1238.

[57] Rahman S, Gain A. Adaptation to river erosion induced displacement in Koyra Upazilla of Bangladesh. Prog in Disaster Sci. 2020;5(20):10055.

[58] Braun M, Saroar M. Participatory action research on climate risk management, Bangladesh. WorldFish, Penang, Malaysia. Studies & Reviews. 2012;39.

[59] Nishat B. Landscape Narrative of the Sundarban: Towards Collaborative Management by Bangladesh and India (English). Washington, DC: World Bank Group; 2019.

[60] Saroar MM. Ecosystem-based adaptation (EbA) for coastal resilience against water related disasters in Bangladesh. In: Leal Filho W, editor. Climate Change Impacts and Adaptation Strategies for Coastal Communities. Springer, Cham; 2018. pp. 187–205.

[61] Vousdoukas MI, Mentaschi L, Voukouvalas E, Verlaan M, Jevrejeva S, Jackson LP, et al. Global probabilistic projections of extreme sea levels show intensification of coastal flood hazard. Nat Commun. 2018;9:2360. doi: 10.1038/s41467-018-04692-w 29915265 PMC6006144

[62] Brown S, Nicholls RJ. Subsidence and human influences in mega deltas: the case of the Ganges-Brahmaputra-Meghna. Sci Total Environ. 2015;27–528:362–374. 25974280 10.1016/j.scitotenv.2015.04.124

[63] Haq SMA, Ahmed KJ. Does the perception of climate change vary with the socio-demographic dimensions? A study on vulnerable populations in Bangladesh. Nat Hazards. 2016;85:1759–1785.

[64] Haque Md A, Rahman D, Rahman Md H. The importance of community based approach to reduce sea level rise vulnerability and enhance resilience capacity in the coastal areas of Bangladesh: a review. J Sustain Sci Manag. 2016;11(2):81–100.

[65] Nandy P, Islam MA. Climate resilient coastal zone development in Bangladesh: participatory governance for common resources management. In: Ramanathan AL, Bhattacharya P, Dittmar T, Prasad MBK, Neupane BR, editors. Management and Sustainable Development of Coastal Zone Environments. Dordrecht: Springer; 2010. pp. 58–72.

[66] Mukul SA, Alamgir M, Sohel MdSI, Pert PL, Herbohn J, Turton SM, et al. Combined effects of climate change and sea-level rise project dramatic habitat loss of the globally endangered Bengal tiger in the Bangladesh Sundarbans. Sci Total Environ. 2019;663:830–840. doi: 10.1016/j.scitotenv.2019.01.383 30738263

[67] CCC (Climate Change Cell). Assessment of Sea Level Rise on Bangladesh Coast through Trend Analysis. Department of Environment, Ministry of Environment and Forests, Government of the People’s Republic of Bangladesh, Dhaka. 2016.

[68] Duarte CM, Losada IJ, Hendriks IE. The role of coastal plant communities for climate change mitigation and adaptation. Nat Clim Chang. 2013;3:961–968.

[69] Islam A, Shaw R, Mallick F. National Adaptation Programme of Action. In: Shaw R, Mallick F, Islam A, editors. Climate Change Adaptation Actions in Bangladesh. Disaster Risk Reduction (Methods, Approaches and Practices). Tokyo: Springer; 2013. pp. 93–106.

[70] Saroar MM, Routray JK. Climate Refugee is not a hoax. But we can avoid it. Empirical evidence from the Bangladesh coast. In: Schmidt-Thome P, Klein J, editors. Climate change adaptation in practice: from strategy development to implementation. John Wiley & Sons; 2013. pp. 283–301.

[71] Uddin MS, Shah MAR, Khanom S. Climate change impacts on the Sundarbans mangrove ecosystem services and dependent livelihoods in Bangladesh. Asian J Conserv Biol. 2013;2(2):152–156.

[72] Sarker SK, Matthiopoulos J, Mitchell SN. 1980s–2010s: The world’s largest mangrove ecosystem is becoming homogeneous. Biol Conserv. 2019;236:79–91. doi: 10.1016/j.biocon.2019.05.011 31496538 PMC6716549

[73] Iftekhar MS, Saenger P. Vegetation dynamics in the Bangladesh Sundarbans mangroves: a review of forest inventories. Wetl Ecol Manag. 2008;16(4):291–312.

[74] Roy S. Climate Change Impacts on Gender Relations in Bangladesh. Springer, Cham; 2019.

[75] Saha SK. Cyclone Aila, livelihood stress, and migration: empirical evidence from coastal Bangladesh. Disasters. 2017;41(3):505–526. doi: 10.1111/disa.12214 27654847

[76] Kelman I, Bayes A, Esraz-Ul-Zannat M. Warning systems as social processes for Bangladesh cyclones. Disaster Prev Manag. 2018;27(4):370–379.

[77] BBS (Bangladesh Bureau of Statistics). District Statistics, 2011: Satkhira. Statistics and Informatics Division (SID). Ministry of Planning, Government of the People’s Republic of Bangladesh, Dhaka. 2013.

[78] Yamane T. Elementary Sampling Theory. New Jersey: Prentice-Hall, Inc; 1967.

[79] Alam GMM. An assessment of the livelihood vulnerability of the riverbank erosion hazard and its impact on food security for rural households in Bangladesh. PhD Thesis, University of Southern Queensland, Toowoomba, Australia. 2016. Available from: https://eprints.usq.edu.au/34160/1/Alam_2016_whole.pdf

[80] Long H, Liu Y, Wu X, Dong G. Spatio-temporal dynamic patterns of farmland and rural settlements in Su-Xi-Chang region: implications for building a new countryside in coastal China. Land Use Policy. 2009;26(2):322–333.

[81] Islam SM, Naher N, Roy N, Mahmud MdK, Hossain MdD, Modak S. Agricultural Adaptation Options against Adverse Effect of Climate Change in Shyamnagar Upazila in the Satkhira District, Bangladesh. Asian J Res in Agricultur For. 2019;2(3):1–12.

[82] Hasanuzzaman M, Hossain M, Saroar M. Diversity and preference of agricultural crops in the cropland agroforests of southwestern Bangladesh. Int J Agric. Crop Sci. 2014;7(7):364–372.

[83] Vinning G, Alam GMM, Das OC, Yeasmin F, Das R. Gross margins for paddy, aquaculture and livestock in Satkhira District, Southwest Bangladesh; In: Adaptive early recovery in water-logged communities in Satkhira District—Phase II. OSRO/BGD/503/WFP. FAO, Dhaka; 2016.

[84] Alam GMM, Alam K, Mushtaq S, Nazirul Md, Sarker I, Hossaine M. Hazards, food insecurity and human displacement in rural riverine Bangladesh: Implications for policy. Int J Disaster Risk Reduct. 2020;43:101364.

[85] Smit B., Wandel J. Adaptation, adaptive capacity and vulnerability. Glob Environ Change. 2006;16(3):282–292.

[86] Adger WN, Dessai S, Goulden M, Hulme M, Lorenzoni I, Nelson DR, et al. Are there social limits to adaptation to climate change? Clim Change. 2009;93(3–4):335–54.

