# Supplementary material for: Climate change adaptation responses among riparian settlements: A case study from Bangladesh
Source: PLoS One. 2022 Dec 7;17(12):e0278605. doi: 10.1371/journal.pone.0278605 (PMC9728924; doi:10.1371/journal.pone.0278605)
Supplement: S2 File — (PDF) [file pone.0278605.s002.pdf]

Climate change adaptation responses among riparian settlements: a case study from Bangladesh

Adaptation strategies	Number of household (multiple option)	Response (%)
Greater emphasis on Aman with supplementary irrigation	5	3
Cultivation of different rice varities (e.g., BRRI-39,49, Bina 7)	4	2.4
Adjusting planting calendars	11	6.6
Adjusting planting techniques	19	11.4
Diversifying crops and varieties	40	24
Vegetables cultivation	22	13.2
Homestead gardening	24	14.4
Livestock rearing	16	9.6
Poultry & Duck rearing	20	12
Family members' migration to other areas, mainly urban areas	6	3.6
Off-farm work(van, auto rickshaw driving etc)	47	28.2
Petty business/small business	42	25.2
No adaptation	2	1.2

Barriers to adaptation	Responses	Percentage
Lack of information on weather and climate change forecast	48	28.8
Lack of money	36	21.6
Lack of knowledge concerning appropriate adaptation to build resilience	56	33.6
Shortage of labour	43	25.8
Lack of water for irrigation	39	23.4
Lack of processing and storage facilities for agriculture products	36	21.6
Lack of market for selling products	34	20.4
Lack of education	35	21
High cost of improved crop varieties	62	37.2
Poor agricultural extension service delivery	60	36
